# P05 A targeted antimicrobial pharmacy technician ward service improves stewardship and patient-centred care and safety of commonly prescribed higher risk antimicrobials in the acute hospital setting

**DOI:** 10.1093/jacamr/dlae136.009

**Published:** 2024-08-23

**Authors:** R Rodger, S Thompson, H Qadri, E Simpson

**Affiliations:** NHS Greater Glasgow & Clyde, Glasgow, UK; NHS Greater Glasgow & Clyde, Glasgow, UK; NHS Greater Glasgow & Clyde, Glasgow, UK; NHS Greater Glasgow & Clyde, Glasgow, UK

## Abstract

**Background:**

Evolving antimicrobial pharmacy technician (AMPT) roles provide opportunity to support antimicrobial stewardship (AS) and patient-centred care in hospital.^1,2^ Documenting antimicrobial stop dates on the electronic prescribing system (HEPMA) improves AS by ensuring patients receive the appropriate treatment duration.^3^ Keeping patients informed and involved^4^ when prescribing higher risk antimicrobials such as aminoglycosides,^5^ fluoroquinolones^6^ and other ‘4C’ agents (co-amoxiclav, clindamycin and cephalosporins) aids early detection and management of adverse effects and drug interactions, supporting treatment optimization and patient-centred care and safety.

**Objectives:**

To develop a targeted AMPT ward service to improve AS and patient-centred care and safety of commonly prescribed higher risk antimicrobials in the acute setting.

**Methods:**

A quality improvement (QI) approach was used to introduce an AMPT service to the Royal Alexandra Hospital inpatient wards. HEPMA reports were used to target AMPT review including: oral antimicrobial treatment courses without a stop date; IV gentamicin prescriptions; and ‘4C’, clarithromycin and fluconazole oral treatment courses. Electronic tools (Microsoft Forms^®^) were developed to support standardization of AMPT patient counselling, interaction management and data collection. ‘Antibiotics it’s OK to Ask’,^7^ fluoroquinolone^6^ and gentamicin^5^ patient information leaflets were provided, and referrals were made to the antimicrobial or ward pharmacist where appropriate. Wards achieving 75% documentation of oral antibiotic stop dates on HEPMA and AMPT interventions were collated as indicator measures of improvement.

**Results:**

AMPT review identified the following. (i) Twenty-three percent (*n*=523) of oral antimicrobials without a stop date were ‘4C’ antibiotics and 9% fluoroquinolones. Thirty-five percent of patients were suitable for counselling; common barriers included cognitive impairment or poor clinical condition. Of those counselled, 18% were unsure antibiotics were prescribed, 64% knew the indication, 27% knew the antimicrobial agent and only 4% the planned duration. Twenty-three percent stated possible side effects, most commonly gastrointestinal upset. (ii) Sixty-nine percent (*n*=142) of fluoroquinolone, clarithromycin and fluconazole oral treatment courses were prescribed concomitantly with potentially serious interacting medications,^8^ 42% (*n*=98) related to QTc prolongation and 15% were fluoroquinolone/multivalent cation interactions. (iii) Sixty percent (*n*=265) of patients prescribed IV gentamicin were suitable for counselling. Seventy-two percent knew gentamicin treated infection but only 12% knew about potential renal and ototoxicity prior to counselling. Twelve months after AMPT service introduction documentation of oral antimicrobial stop dates improved from baseline in 94% (*n*=18) (Table 1), and the 75% target was achieved in 22% of targeted wards, respectively (Figure 1).

**Conclusions:**

A targeted AMPT ward service resulted in improved AS and patient-centred care and safety of commonly prescribed higher risk antimicrobials in the acute hospital setting.

**Table 1.** Percentage of oral antimicrobials with a documented stop date on HEPMA

	Baseline, median, % (*n*) (April–December 2022)	Post-change, median, % (*n*) (January 2023–January 2024)	Target, %
RAH medical wards			
MW1	43 (358)	63 (289)	75
MW2	69 (143)	68 (277)	75
MW3	61 (207)	67 (241)	75
MW4	54 (216)	58 (362)	75
MW5	49 (270)	72 (406)	75
MW6	52 (244)	81 (460)	75
MW7	53 (171)	78 (323)	75
MW8	62 (156)	68 (278)	75
MW9	51 (199)	70 (322)	75
RAH surgical wards			
SW1	31 (244)	53 (233)	75
SW2	49 (122)	71 (180)	75
SW3	53 (103)	93 (121)	75
SW4	60 (102)	69 (167)	75
SW5	43 (195)	57 (287)	75
SW6	27 (60)	60 (108)	75
SW7	48 (65)	70 (147)	75
SW8	48 (168)	59 (214)	75
RAH older people services/stroke wards 1,2,3,4,5	75 (408)	77 (894)	75

**Figure 1.**
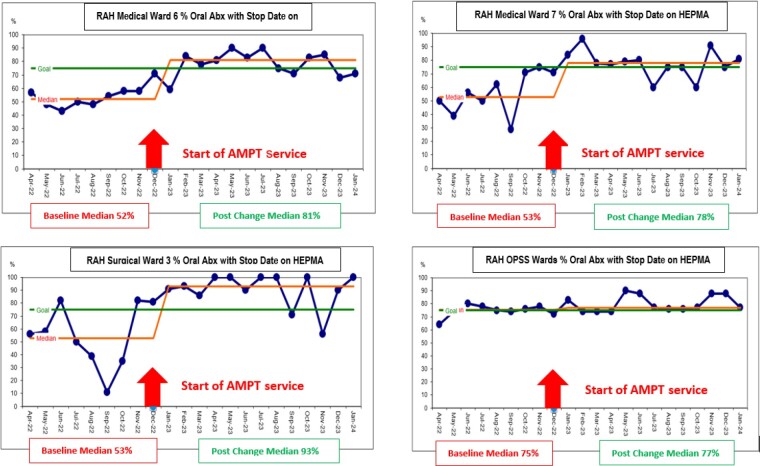
Achievement of 75% target in targeted wards.
